# East Texas Medico-Chirurgical Association

**Published:** 1906-01

**Authors:** J. B. Ramsey

**Affiliations:** Secretary; Alto, Texas


### Society Notes.

### East Texas Medico-Chirurgical Association.

ALTO, TEXAS, December 16, 1905.

DEAR DOCTOR: The subject of organization of the medical fraternity is now agitating the minds of the members of our profession more than ever, and we are looking forward for great results, that shall redound to great good to the individual doctor, and the general public, and be the means of raising the standard of medical teaching, and elevating the healing art in the minds of the people.

Under the new order of things, as promulgated by the American Medical Association, it has been thought by some that Independent Medical Societies had served their purpose, but at the last meeting, in Palestine, of the East Texas Medico-Chirurgical Association, it was found to be the opinion of its members that it was not yet time to discontinue our organization, and by a unanimous vote it was perpetuated until such time as an official district association could be organized.

Our Association was never in better working order, and every member is urged to put forth greater efforts to make it more pleasant and profitable, and it is sincerely hoped that you will lend us a portion of your valuable time to attend its meetings, and, if possible, to contribute a paper or report a clinical case at our next meeting, which will be held in Palestine on the fourth Thursday and Friday in May, 1906.

I am instructed to furnish you with a copy of our By-Laws, which I herewith enclose. Please notify me at your earliest convenience the subject of your paper.

With kindest regards, I beg to remain,

Yours truly,

J. B. RAMSEY, Secretary.

